# Progress towards unique patient identification and case-based surveillance within the Southern African development community

**DOI:** 10.1177/14604582221139058

**Published:** 2023

**Authors:** Kerensa Govender, Lawrence Long, Jacqui Miot

**Affiliations:** Faculty of Health Sciences, University of the Witwatersrand, Johannesburg, South Africa; Health Economics and Epidemiology Research Office (HE^2^RO), Wits Health Consortium, University of the Witwatersrand, Johannesburg, South Africa; Faculty of Health Sciences, University of the Witwatersrand, Johannesburg, South Africa; Health Economics and Epidemiology Research Office (HE^2^RO), Wits Health Consortium, University of the Witwatersrand, Johannesburg, South Africa; Department of Global Health, Boston University School of Public Health, Boston, MA, USA; Faculty of Health Sciences, University of the Witwatersrand, Johannesburg, South Africa; Health Economics and Epidemiology Research Office (HE^2^RO), Wits Health Consortium, University of the Witwatersrand, Johannesburg, South Africa

**Keywords:** case-based surveillance, health-systems strengthening, linkage, patient tracking, unique patient identifier

## Abstract

Population mobility makes patient-tracking and care linkage in the South African Development Community (SADC) challenging. Case-based surveillance (CBS) through individual-level clinical data linked with a unique patient-identifier (UPI) is recommended. We conducted a mixed-methods landscape analysis of UPI and CBS implementation within selected SADC countries, this included: (1) SADC UPI implementation literature review; (2) assessment of UPI and CBS implementation for high HIV-prevalence SADC countries; (3) UPI implementation case-study in selected South African primary healthcare (PHC) facilities. Research into CBS and UPI implementation for the SADC region is lacking. Existing patient-identification methods often fail and limit patient-tracking. Paper-based records and poor integration between health-information systems further restrict patient-tracking. Most countries were in the early-middle stages of CBS and faced UPI challenges. Our South African case-study found that the UPI often goes uncaptured. Difficulties tracking patients across prevention and care cascades will continue until a functional and reliable UPI is available.

## Background

In the wake of any health threat, health systems at the national and international level are often tested^1^ with a pertinent example being the current COVID-19 pandemic. The need to strengthen fragile health systems, especially in low-income and resource-constrained countries is driven by the recognition that weak health systems impede attainment of global and national targets, and are insufficiently prepared to respond to crises.^2^ The HIV response in the Southern African Development Community (SADC) is one example of a public health-system strengthening initiative to improve access to care for people living with HIV (PLHIV).^3^ Countries, such as South Africa (SA), with the highest burden of HIV infection globally, have made significant progress in making HIV care more accessible through universal prevention, testing, and treatment policies.^4^ Despite this, challenges in linking patients to HIV care persist, mostly due to losses of HIV diagnosed clients at pre-ART (anti-retroviral therapy) stages in Sub-Saharan Africa.^5^ A systematic review within the region found that a median of 68% (range 14%–84%) of patients eligible for ART actually went on to initiate treatment within the observed study period at the clinic of diagnosis.^6^ As a result, many Sub-Saharan countries have missed key milestones, such as the 90–90-90 targets set by The Joint United Nations Programme on HIV/AIDS (UNAIDS).^3,5^ The implementation of a unique patient-identifier (UPI) is recommended to reach these goals through the creation of a strategic health information system (HIS) to identify and track PLHIV; additionally the UPI strengthens other health system service areas through linkage, therefore creating a more person-centred and integrated approach to healthcare.^7^

A HIS which supports patient level data collection across multiple sources is important for improving consistency of care, treatment adherence and ultimately health outcomes, especially for more vulnerable populations such as PLHIV.^3^ There is however, an overall paucity of reliable health-system surveillance data on the African continent.^8^ The mobile nature of the population also makes HIV testing and long-term care challenging.^9^ A limitation of commonly used facility-level surveillance programs is the inability to uniquely identify a patient across the health system, often resulting in inaccurate interpretation of surveillance data.^10^ In 2017 the WHO released a consolidated set of HIV patient monitoring guidelines, advocating for a more person-centred approach to health information and reporting through case-based surveillance (CBS) by means of a UPI.^11^ A UPI is recommended to create individually linked de-identified longitudinal medical records enabling accurate monitoring, evaluation and subsequent service improvements on a continuous basis.^12^ The UPI can take the form of a numeric, alphanumeric or biometric anonymous identifier(lacks identifying data) and facilitates data-linkage if systems are sufficiently integrated and connected to the internet.^7,11^

Most resource limited countries recognise the need to shift away from aggregated monitoring within their HIV care-cascade towards a person-centred, second generation HIS which supports CBS by tracking individual-level clinical data with a UPI.^12,13^ Such systems facilitate quality HIV care which requires ongoing monitoring of clinical outcomes, medication adherence, and long-term retention in care.^14^ Furthermore the ability to register and track patients through UPI linked datasets grows even more critical as countries move towards National Health Insurance (NHI) implementation.^15^ Unique identification of individuals is important for monitoring of progress towards NHI, measured through two components requiring disaggregated and accurate data, these are: (i) the proportion of the population accessing essential health care services and (ii) the proportion of the population for which health costs take up a large part of household expenditures.^16^ The US President’s Emergency Plan for AIDS Relief (PEPFAR), which supports efforts for HIV epidemic control in several African countries, has therefore made the implementation of UPI based CBS an important public-health policy consideration within supported countries.^17^ This investigation seeks to provide a landscape analysis of progress towards CBS implementation with a UPI within selected SADC countries.

## Methods

We used a mixed-methods study design with three activities: (1) a literature review of existing peer reviewed evidence on patient-tracking and UPI implementation, as well as underlying drivers behind the need for a UPI within the SADC region; (2) a detailed assessment of progress towards national CBS implementation through UPI-based patient tracking within relatively high HIV prevalence SADC countries; and (3) a case-study of UPI implementation at selected South African primary healthcare (PHC) facilities.

### Activity 1: Literature review

The literature search was conducted in PubMed using keywords specific to health-systems and patient identification, as well as SADC country names. All possible synonyms, and keyword combinations were then generated from the main key words using the OR operator and included in the search through Medical Subject Headings (MeSH) in PubMed. Detailed search terms are provided in Table 1.

**Figure 1.**
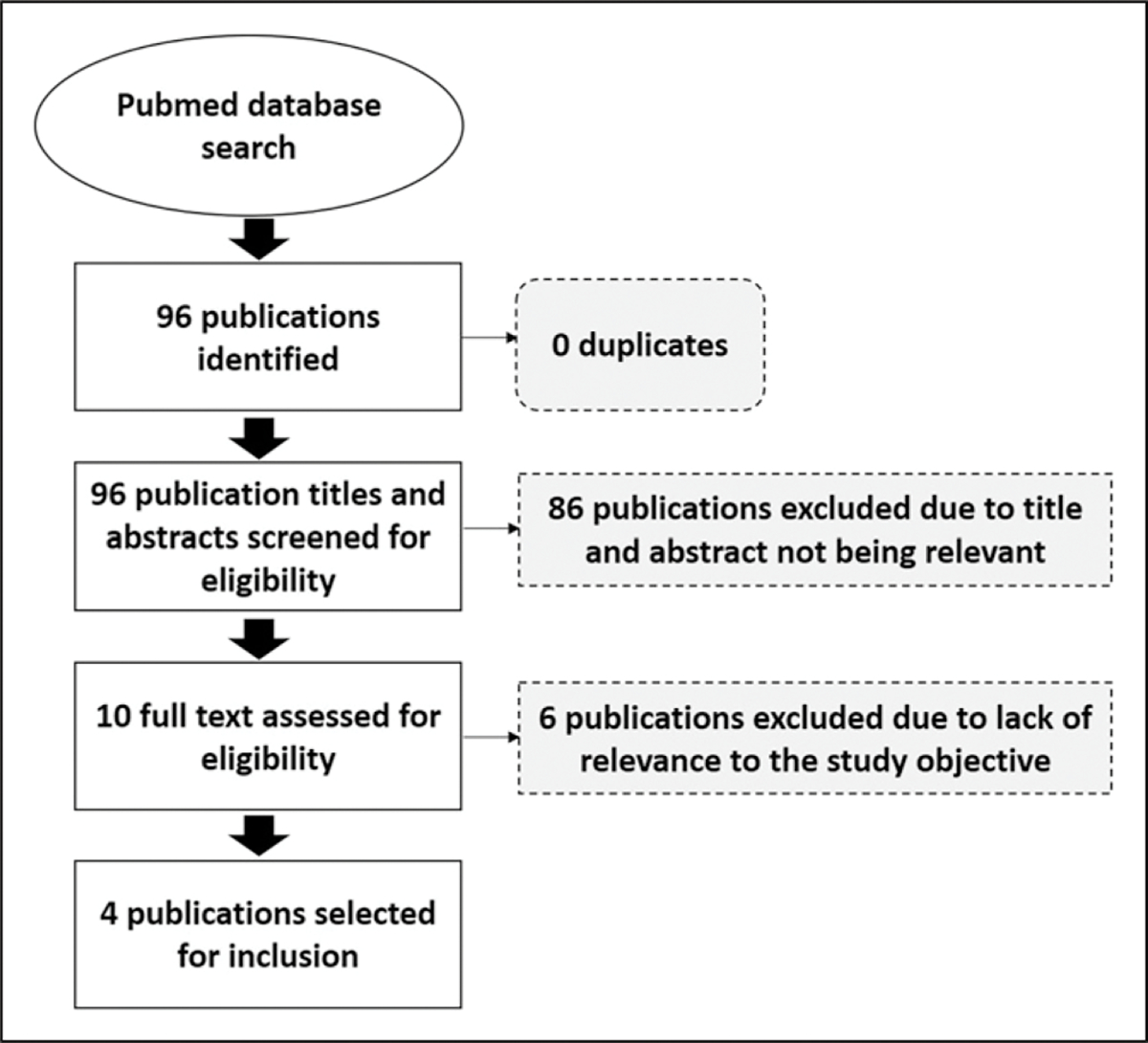
PRISMA flow diagram for the study selection process.

SADC based studies, published in English between 2009 to 2019, with a study population of adults (≥18 years) or adolescents (10–19 years) were considered for review. For inclusion, studies were required to address the subject of patient identification and/or patient tracking. All three authors were involved in title and abstract screening, as well as the full-text assessment for eligibility and inclusion. To minimise screening and selection bias each author independently screened the titles and abstracts of the 96 eligible publications. Conflicts were discussed until a consensus was reached. Full text assessments followed the same process. Screening and selection were conducted on Rayyan (https://rayyan.qcri.org), a tool for systematic reviews. Refer to Figure 1 for further details.

One author then read the publications selected for inclusion and completed a data extraction form requiring information on: author(s), study setting, main study objectives, study population, study design and study findings. A descriptive narrative synthesis was used for data synthesis.

### Activity 2: Assessment of progress towards national CBS implementation through patient tracking with a UPI within relatively high HIV prevalence SADC countries

Country selection for Activity two was informed by the 2018 UNAIDS country level HIV prevalence estimates due to the critical role of CBS in the delivery of quality HIV care.^18^ Countries reporting an HIV prevalence equal to or above the SADC region average (estimated to be 10,5% (2018)) met our inclusion criteria. Botswana, Eswatini, Lesotho, Mozambique, Namibia, South Africa, Zambia and Zimbabwe met this criterion. A toolkit, informed by the WHO stages of development.^11^ for the transition from name and paper-based individual records to an electronic record coded with a UPI, was developed to guide data collection. The toolkit is premised upon the six essential elements of the transition to CBS, (i) Person identification: assigning and using unique-identifiers; (ii) Investing in databases and interoperability; (iii) Confidentiality and security; (iv) Data analysis, quality and use; (v) Transition from paper to electronic systems and (vi) Sustainability of programme improvements.^11^ The latest PEPFAR Country Operational Plans (PEPFAR-COP) for each country was the main source for this activity. Findings from these documents were supplemented with relevant literature, found through a purposive internet-based search using relevant key words, or through conversation with an in-country contact, if available. Data collection occurred between June 2019 and March 2020 and represents each countries status up to March 2020.

### Data analysis

We used the WHO’s three broad stages of CBS development as specified for each of the six essential elements of the transition to CBS to classify country-level progress, namely (i) Early: Switch from name-based records to UPIs associated with a single individual in a paper-based HIS; (ii) Middle: The widespread use of unique-identifiers and the deployment of an electronic data system with a mixture of online and offline elements; and (iii) Advanced: UPI linked datasets in a fully online electronic HIS linked across services, facilities and with community care.^11^ We reviewed all six elements required for CBS implementation to identify gaps in progress (Table 2).

### Activity 3: A descriptive case study of UPI implementation at selected SA clinics

Our study population consisted of adult patients (≥18 years) accessing care at four public sector PHC facilities within South Africa’s Gauteng province. At two facilities we examined these selected conditions/diseases: hypertension (diagnosis and treatment), diabetes (diagnosis and treatment), and tuberculosis (TB) (diagnosis and treatment, excluding drug resistant TB). At all of the facilities we examined HIV (diagnosis and treatment). Data collection for this study was approved by the Human Research Ethics Committee of the authors university. We used a sample size of 75 medical-records per disease cohort at each PHC facility to conduct a retrospective medical-record review of paper-based health records, which facilities primarily rely on alongside existing electronic HISs such as TIER.net for HIV.^19^ This was not meant to be a powered sample but rather meant to be descriptive therefore sample size was feasible given existing work being conducted at the site. Data collection occurred between December 2018 and September 2019. For each study subject, we made a note of the primary disease that the patient presented for initially, as well as which identifiers (i.e. Name, Surname, National ID, Passport number etc.) had been captured onto their medical record. Each study subject was assigned a study-ID; all data collection occurred under this study-ID without any patient identifying information. Data was entered into a REDCap database; a secure, web-based platform designed to support research data collection.^20^ Stata Statistical Software (Release 15. College Station, TX: StataCorp LLC) was used for data-analysis. Descriptive statistics were generated to understand the collection of identifiers per site and per disease cohort across sites. We specifically focused on the SA national identity (ID) number and passport/foreign passport numbers which are critical for the UPI based Health Patient Registration System (HPRS) implementation.^21^

## Results

### Activity 1: Literature review

From the 96 publications identified, four studies conducted in three SADC countries, namely Mozambique, South Africa and Zambia met our inclusion criteria. The review revealed a gap in the literature on UPI implementation, patient-tracking and identification systems for the SADC region. Articles included in the review focused on two categories: (1) existing patient identification methods and (2) patient identification and tracking of long-term health outcomes. Refer to Table 3.

### Existing patient identification methods

Two studies described existing patient identification methods and challenges.^22,23^ Patient identification within and between health facilities is a challenge in much of Sub-Saharan Africa with existing methods of identification not unique to a patient.^23^ Furthermore many health clinics in the region still use identifiers such as patient name, date of birth, government ID, phone numbers and facility specific file numbers making it difficult to identify and/or follow a patient across health facilities.^22^ The use of patient name gives rise to confidentiality concerns and is subject to spelling errors resulting in poor data linkage and duplicate or incomplete records.^23^

### Patient identification and tracking of long-term health outcomes

Four articles reported on patient identification and tracking of long-term health outcomes.^22–25^ Program specific reporting indicators in resource limited settings have historically been calculated manually on a monthly basis from a health facility’s paper-based registers, and then aggregated at the district, provincial and national-level.^25^ UPIs strengthen fragmented health services by linking data held within facilities and enabling information flow across the healthsystem.^23^ This creates a complete health-record which is vital for monitoring site performance and informing national planning, as well as the effective delivery of care over time.^25^ A study conducted in the Western Cape province (WCP) of South Africa noted that despite the importance of analysing longitudinal health outcomes for effective monitoring and evaluation, such data is often incomplete due to population mobility and silent transfers between facilities.^22^ Patients in this study were assigned one UPI across all services, which showed great potential for the linking and tracking of adolescents living with HIV and their health outcomes due to improved systems integration.^22^ A Zambian study implemented a digital-biometric (fingerprint) identification system to create a unique and secure patient identifier which provides integrated and real-time data.^23^ Although there were concerns about the provision of fingerprints, results indicated that the system was feasible and effective at uniquely identifying female sex workers, a typically hard to reach, stigmatized and highly mobile population.^23^ Another South African study similarly highlighted the advantage of biometric identification in clinical trial participants; results showed that such systems can store and provide accurate and secure patient information timeously without requiring identifying documents.^24^

### Activity 2: Assessment of progress towards national CBS implementation through patient tracking with a UPI within relatively high HIV prevalence SADC countries

**Figure 2.**
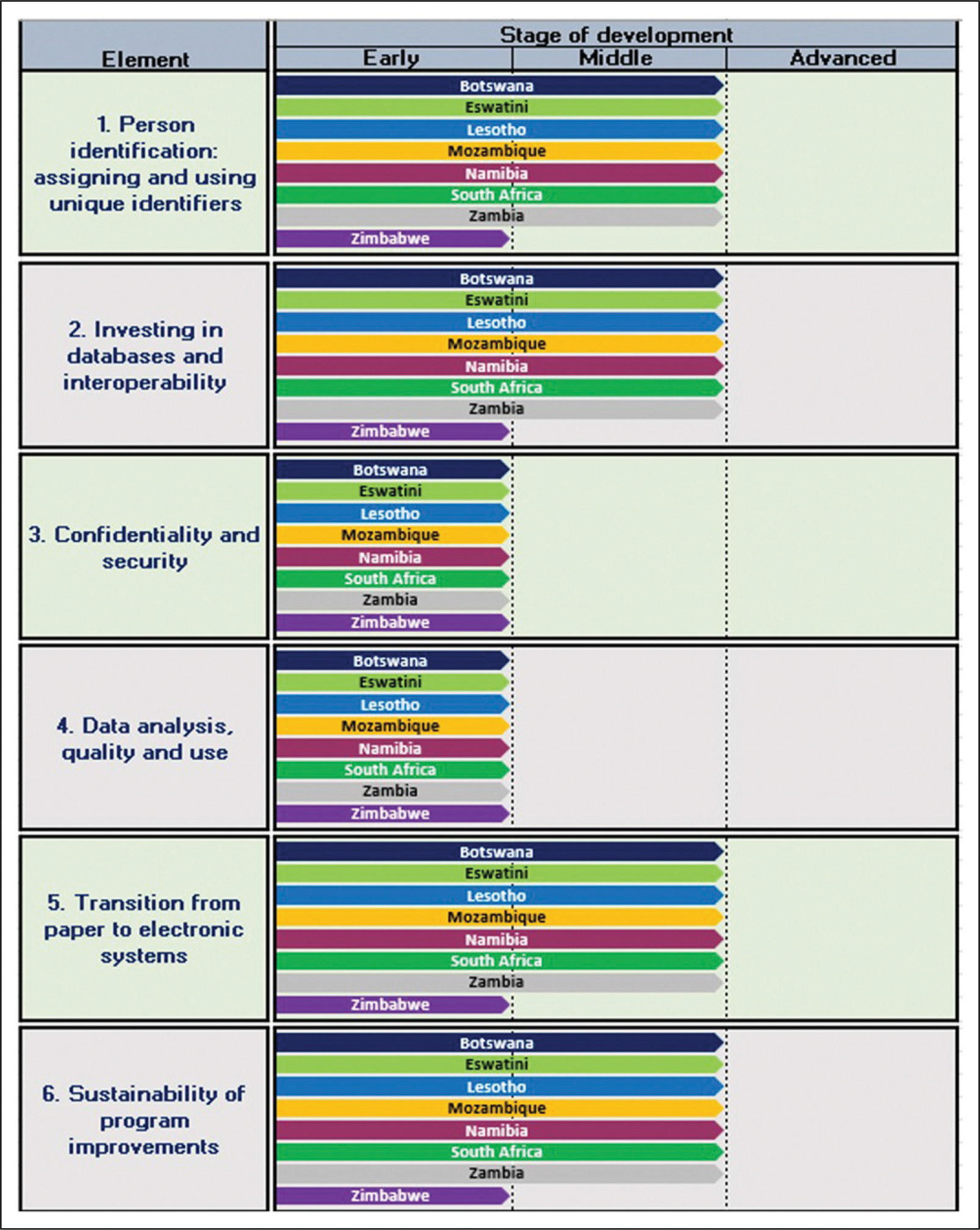
Comparison of results by the seven key elements for the transition to case-based surveillance across the selected countries.

Eight countries met the inclusion criteria for this analysis (Botswana, Eswatini, Lesotho, Mozambique, Namibia, South Africa, Zambia and Zimbabwe). Figure 2 presents results for each of these countries, classified by stage of development across the six essential elements of the transition to CBS. Full details of country specific assessments have been attached with this paper (see Additional file 1). All countries, with the exception of Zimbabwe, appear to have reached the middle-stages of development for four out of the six essential elements; results suggest that UPI implementation challenges currently limit HIS integration and patient-tracking capabilities which may in turn prevent the region from moving towards a truly second-generation HIS. Findings are reported below and point to key areas requiring attention. Further details on the results of this assessment have also been provided in appendices A–H.

#### Patient identification.

With the exception of Zimbabwe, the seven remaining countries were reported to be in the process of rolling out a national HIS which made use of both name and paper-based health records (early stage criteria) while simultaneously beginning to implement UPIs at facility level (middle stage criteria).^26–32^ Eswatini makes use of the national ID number as the UPI while South Africa aims to implement a 10-digit system generated UPI known as the Health Patient Registration Number (HPRN) linked to the SA ID number or SA/foreign passport number; other countries report using a system generated number, or smartcard number as a UPI. UPI implementation within these countries appears to focus mainly on PLHIV. PEPFAR Namibia is however working on introducing a UPI across disease areas and South Africa is in the process of rolling out the HPRS with UPI linked longitudinal health records across disease areas.^21^ There are however persistent challenges with UPI implementation and use in all countries. Mozambique, for example, is reported to have a national Electronic Patient Tracking System (EPTS) which is reliant on a UPI at most PEPFAR supported facilities but is dealing with issues of multiple UPIs being assigned to a patient within a facility.^29^ Zimbabwe was still in the process of developing a UPI at the time of conducting this assessment and therefore rated as early stage.^33^

#### Investing in databases and interoperability.

All countries, besides Zimbabwe, had met the middle-stage criteria for this element, having rolled out an Electronic Health Information System (eHIS) to most PEPFAR supported and national facilities. Paper-based health systems do however still exist alongside the eHIS within these countries. Integration between the two systems is manual requiring a data-capturer for data transfer; staff shortages and limited training may result in incomplete and inaccurate electronic records.^14,26,30,33–37^ Connectivity issues further inhibit integration between the two systems as seen in the more rural areas of Zambia, where information is captured on SmartCare forms which are then transported to an area with internet connectivity to be captured into Smartcare, the HIV system.^37^ Interoperability between various standalone eHISs is another challenge. For example, the Patient Information Management System (PIMS) in Botswana has not been integrated with the laboratory system (Integrated procurement management system) and requires a manual transfer of results into PIMS;^26^ similarly the South African HIV health system TIER.Net also requires a manual transfer from other key databases, such as the national laboratory system.^31^ Furthermore, South Africa’s eHIS (HPRS) which is reliant on a system generated UPI or HPRN (linked to a patient’s SA ID number or SA/foreign passport number) has not reached scale as yet and isn’t fully integrated with the facility specific Tier.net system.^21,31^ Eswatini, Lesotho, Mozambique, Namibia and Zambia also face interoperability challenges between their standalone eHIS, and other key databases such as the laboratory and dispensing systems.^27–29,32,38^ Zimbabwe is at the early stage of development due to a limited number of facilities with access to the country’s Electronic Patient Management System (EPMS) with most facilities solely reliant on paper based records.^33^

#### Confidentiality and security.

The literature suggests that all countries were at the early stages of development for this element with personal information such as name and surname still captured into the eHIS, despite the existence of a UPI.^26–32,39^ In Zambia, although records are coded with the UPI from the Smartcare card, personal data is still collected in case the card is lost to enable access to the patient record.^40^ Similarly, in Swaziland records on the Client Management Information System are coded with a UPI and personal content.^34^ Paper based records are most likely stored at facility level with restricted access for all countries.

#### Data analysis quality and use.

All countries appeared to be at the early stage of data analysis and use capabilities. This seems to be due to poor integration between their eHIS and other health-systems, as well as challenges with duplicate identifiers, clinic specific identifiers and incorrect capturing of the UPI into the eHIS, as experienced with the unique ART number in Namibia; all of which has made patient tracking and the creation of longitudinal records difficult.^25–39^ All countries remain reliant on data officers, or implementing partners, to transfer data from paper records into various eHIS.^25–39^

#### Transition from paper to electronic systems.

Due to the limited number of facilities accessing the ePMS in Zimbabwe the country was considered to still be in the early stages of CBS development.^33,39^ The remaining countries were classified as having reached the middle stages of development as reports suggest that an eHIS has been implemented alongside paper-based records in the majority of PEPFAR supported facilities; offline upload of information into the electronic system is still required.^26–32,41^

#### Sustainability of programme improvements.

With the exception of Zimbabwe, all countries appear to have reached the middle stages of development for this element. This is due to progress in patient tracking within a facility using the eHIS; challenges related to capturing and assigning of a UPI, as well as a lack of system integration, does however mean that tracking is largely still reliant on paper-based systems and personal information.^26–32,41^

### Activity 3: A descriptive case study of UPI implementation at selected SA clinics

**Figure 3.**
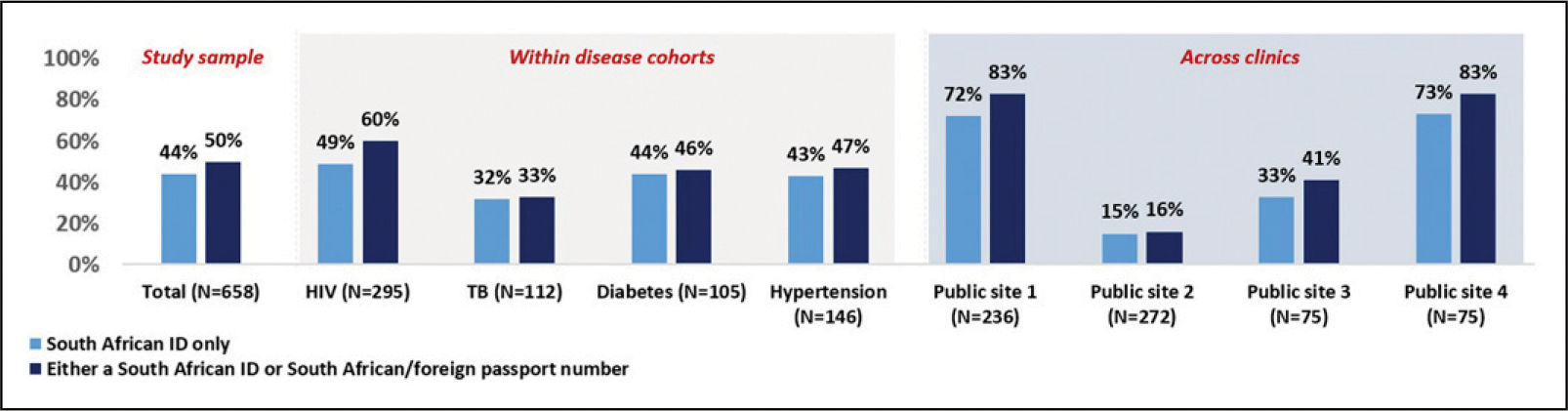
Proportion of files with a national ID and/or passport/foreign passport identifier captured for the study sample, and within each disease cohort.

Results indicate that the recommended identifier (SA ID or SA passport/foreign passport) required for HPRS implementation and the generation of the UPI or HPRN^21^ is often not captured at our selected clinics. Under half (44%, *N* = 289) of all files sampled had the SA ID number while 50% (*N* = 332) had either a SA ID or SA passport/foreign passport captured, refer to Figure 3, with results varying across clinics and disease cohorts.

HIV files were more likely to have the recommended identifier, with 60% of all HIV files having either the SA ID or SA passport/foreign passport details on them. The TB cohort had the lowest proportion (33%, *N* = 37) of files with these identifiers captured, followed by diabetes and hypertension files (46% and 47% respectively). None of the files in our sample had the HPRN recorded.

**Figure 4.**
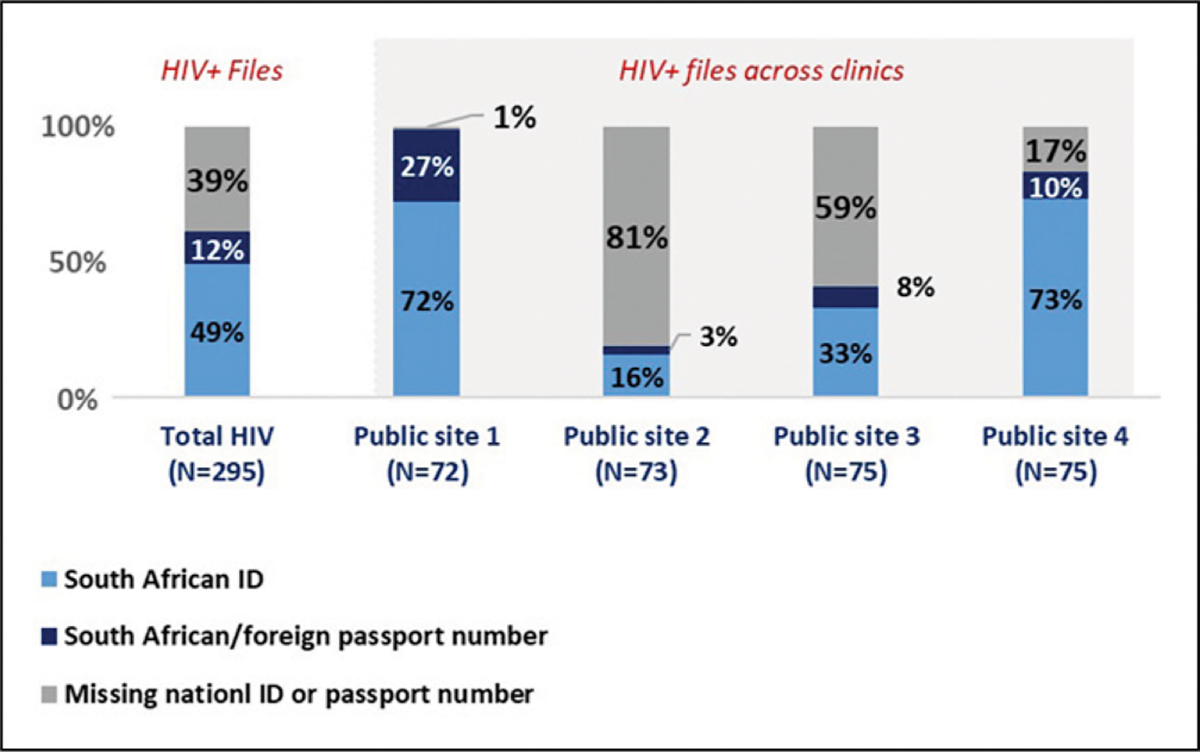
Results of an analysis into HIV files and identifiers by study site.

Further analysis into HIV files (Figure 4) revealed process variances across clinics, Public site one and Public site four were more likely to capture either the SA ID number, SA passport number or foreign passport number (99% and 83% of all files respectively). In comparison just 19% of HIV files at Public site 2 and 41% of HIV files at Public site three had either a SA ID number, SA passport number or foreign passport number.

## Discussion

This landscape analysis sought to explore progress towards CBS with a UPI, in selected SADC countries. Our research revealed that while most countries remain in the early-middle stages of the move to a second-generation HIS, further development is hampered by UPI related challenges.

We found that patient monitoring and tracking, which is critical to improving the consistency of care in PLHIV and others, is reliant on the integration of all health systems; this integration is in turn dependent on the consistent assignment, recording and use of a UPI. Despite this, all countries which had rolled out an eHIS faced similar challenges with UPI implementation, this included multiple UPIs being assigned to one person, lost or forgotten system generated identifiers often linked to a smartcard as well as improper and inconsistent recording of the UPI by health personnel. Additionally UPI implementation has varying degrees of financial and technical requirements such as the rollout of a smartcards with smartcard readers or biometric (fingerprint/optical) readers all of which depend on reliable connectivity to function optimally.^7^ Prior research also points towards UPI implementation challenges as an obstacle to HIS evolution amongst African countries.^35,42^ Poor linkage between clinics, insufficient training of staff responsible for capturing unique-identifiers, and staff shortages contribute to these challenges.^43^ Literature reviews by Odekunle et al. as well as Hoque and Sajedur noted similar challenges to health technology adoption within Sub-Saharan Africa which included the high costs of procurement and maintenance of the HIS, poor systems integration, lack of financial incentives, limited computer skills as well as unreliable electricity-supply and internet connectivity.^44,45^ Furthermore, HIV programme monitoring has been reported to be especially challenging due to both its scale and multiple data constraints, these include the lack of reliable information, and the continued use of paper registers which are often incomplete and lack unique-identifiers.^43^ This is consistent with the findings from our South African case study which noted that the recommended UPI may often not be recorded on paper-files and therefore not transferred into the HPRS and Tier.net systems.

Paper-based records are still preferred despite the presence of an eHIS. These were often implemented alongside the eHIS; and required a data-officer to capture information from paper files into the eHIS. Previous studies similarly noted that paper-based records are the primary method for recording and tracking patient data in the region,^14^ and data entry from paper-based forms into the eHIS often falls upon the implementing-partners (IPs).^37^ In addition to the preference for the paper-based records, we noted that the collection of personal identifiers into the eHIS also hampers CBS progress. Holmes et al.^17^ similarly found that the lack of a UPI and poor levels of privacy and patient confidentiality acted as barriers to CBS progress and implementation.

Our country specific analysis suggests that slow UPI implementation as well a preference for paper-based records, may perpetuate a reliance on aggregated monitoring systems. Similarly other studies report a reliance on manual data collection and aggregation for monthly reporting.^43,46^ A previous study on surveillance mechanisms on the African continent concluded that systems which measure, rather than aggregate and estimate disease burden over time require prioritization.^8^ Another report noted that despite the importance of data collection and monitoring of PLHIV for care outcomes, countries in the region lack comprehensive data and rely on aggregated datasets; disaggregation needed to be prioritised.^3^ Our findings point towards the lack of UPI based integration between various stand-alone health systems, such as the eHIS and the laboratory system, as also having an influence on the manner in which data is reported and analysed.

Finally, we found that all countries included in this analysis had limited UPI based integration across their many stand-alone health-systems. This may be due to poor UPI implementation or other technical factors such as poor connectivity. An earlier study identified the UPI as an important step in the integration of various stand-alone HISs and the creation of longitudinal health records for patient monitoring and tracking.^12^ Systems integration has previously been noted as a challenge for SADC countries, resulting in incomplete viral-load reporting with some countries.^3^ At present UPI implementation and systems integration has been prioritised for PLHIV within the selected PEPFAR supported countries in our study, with a few exceptions such as the HPRS in South Africa which is said to run across disease areas. Beck et al.^12^ highlighted the importance of integration across services as PLHIV who also have non-communicable diseases (NCD’s) will need to be tracked, and such integration may contribute to the development of services for other chronic conditions in resource limited countries. The WCP in South Africa emerged as a leader in CBS implementation. Each patient is assigned a UPI and has a single health record with information incorporated from HIV, TB and antenatal services.^7^ Additional data sources include laboratory and dispensing data which is automatically incorporated into the system through integration with these databases.^47^

There are several limitations to this study which indicate the need for further exploration of this subject area. One limitation is the lack of recent literature reviewing progress of UPI implementation and challenges within the SADC region. Additionally, this study did not consider databases outside of PubMed. We were extremely reliant on the PEPFAR-COP reports for country specific information. Other data sources included studies carried out between three to 5 years ago, which may contain information which is not currently relevant. While some in-country experts (program managers and researchers) were consulted, a further limitation is the lack of external input. Future research should seek to understand the progress made in addressing the challenges of UPI implementation and identify barriers in the progression towards CBS by eliciting the viewpoints of health workers and other experts in the field. Future work may also focus on the post-implementation aspects of having a functional UPI in place and could include an in-country analysis into the laws and regulations surrounding data privacy and protection which govern the manner in which patient data is shared and used, as well as a discussion on additional concerns around any risk of re-identification through linkage with other data sources.^48,49^

## Conclusion

This study sought to provide a landscape analysis of advancement towards CBS and individual health-records linked with a UPI within the SADC region. The UPI was found to be critical for systems integration and patient monitoring and the creation of stronger HISs. Until a fully functional and reliable UPI is in place difficulties tracking patients across prevention and care cascades will continue. We have shown that progress towards CBS was lagging in all countries at the time of this study, consistently hampered by a preference for paper-based records and poor implementation of the UPI, underscoring a need for increased policy efforts and support to address this gap.

## Supplementary Material

Supplement

## Figures and Tables

**Table 1.** Key search terms for PubMed database.

Category	Search term combined with AND

Health systems and patient identification	“Patient identification Systems”[Mesh] OR identification system, patient OR identification systems, patient OR patient identification system OR system, patient identification OR systems, patient identificACTation OR patient tracking OR patient trackings OR tracking, patient OR trackings, patient
SADC country	Angola[MeSH terms]) OR Botswana[MeSH terms]) OR Comoros[MeSH terms]) OR democratic republic of the Congo[MeSH terms]) OR Swaziland[MeSH terms]) OR Lesotho[MeSH terms]) OR Madagascar [MeSH terms]) OR Malawi[MeSH terms]) OR Mauritius[MeSH terms]) OR Mozambique[MeSH terms]) OR Namibia[MeSH terms]) OR Seychelles [MeSH terms]) OR South Africa[MeSH terms]) OR United Republic of Tanzania[MeSH terms]) OR Zambia[MeSH terms]) OR Zimbabwe[MeSH terms]

**Table 2.** A description of key elements for CBS and their stages of development

CBS element	Stage of development	Description of stage of development

Person identification: Assigning and using unique identifiers	Early	Name-based record and/or aggregate data based on services, not people (tally sheets)
	Middle	Unique identifiers at facility level
	Advanced	Programme or national unique identifiers, people-centred health record systems
Investing in databases and interoperability	Early	Low-cost paper based record system/Traditional stationary costs
	Middle	Facility-based electronic data systems/Basic computer/ Open-access software
	Advanced	Fully interoperable data system, linkage of information from multiple sources. Linkage with vital statistics, migration data. Useful for tracking individuals lost to follow up, etc
Confidentiality and security	Early	Name-labelled paper files retained by the individual or kept under lock and key at facility
	Middle	Records coded with unique identifiers without personal content
	Advanced	National system with health record data protected by law. Limited and enforced data access control
Data analysis, quality and use	Early	Data officer transfers data from paper record into electronic health record or register, regular data quality reviews
	Middle	Programme or central-level analysis of data and creation of management dashboards, and other data analysis and reporting tools
	Advanced	Local analyses of care and programmatic capacity. Standardized dashboards, data visualization and reports. Individual care facilitated by ease of data access, aggregation and review. Regular use of data for decision-making at individual, facility, programme and national levels
Transition from paper to electronic systems	Early	Paper based record system. Records retained at facility or by individual
	Middle	Offline electronic upload of data. On- or offline data access
	Advanced	Fully online systems used across facilities, in community care. Links services within facility and across facilities
Sustainability of program improvements	Early	Patient monitoring is only system in place to track individuals over time. Challenging to link individual data within and between facilities
	Middle	Limited ability to track individuals within a facility. Appointment scheduling, follow up within a facility. Within facility linkage of individual information from clinic to lab and pharmacy
	Advanced	Individual records updated in real-time with clinical, lab, pharmacy and other data. Person-based records linked with death registry data

Source: World Health Organization. Consolidated Guidelines on Person-centred HIV Patient Monitoring and Case Surveillance., 2017.

**Table 3.** Characteristics of studies included in the review.

Authors	Country	Main study objectives	Study population	Study design	Key findings

Lambdin et al., 2012	Mozambique	To assess the completeness and reliability of electronic patient tracking systems (EPTS) used in 16 HIV care and treatment clinics in Manica and Sofala provinces of Mozambique	Adult (≥15 years of age), ART- na¨ıve patients receiving HIV treatment at the selected facilities	Quantitative, cross-sectional analysis to assess the completeness and reliability of data	The electronic patient tracking systems for HIV treatment programs in Manica and Sofala provinces of Mozambique had high levels of completeness and reliability justifying their use
Harichund et al., 2013	South Africa	To report the development and feasibility of a digital, fingerprint-based participant identification method to prevent co-enrolment at multiple clinical trial sites in KwaZulu Natal province	Women enrolled in HIV related trials at seven South African medical research council (MRC) - HIV prevention research unit (HPRU) sites	Pre/Post intervention observation and analysis	The biometric verification system is a novel approach to prevent participant co-enrolment in multiple HIV prevention clinical trials
Wall et al., 2015	Zambia	To determine the feasibility of implementing an electronic-fingerprint linked data capture system in Zambia. Determine the acceptability of this system among a key HIV risk group: Female sex workers (FSWs)	Female sex workers accessing four clinics in Ndola, Zambia	Pre/Post intervention qualitative and quantitative analysis	Implementation of an electronic fingerprint-linked patient tracking and data collection system was feasible in this relatively resource-limited setting and was acceptable among FSWs in a clinic setting
Davies et al., 2017	South Africa	To evaluate long-term outcomes in HIV-infected adolescents after transfer by linking data from four international epidemiology databases to evaluate AIDS Southern Africa (IeDEA-SA) cohorts in the western cape province (WCP) with department of health (DoH) data	All adolescents on ART if they had a valid WCP DoH folder number and were recorded as transferred out between 10 and <20 years of age from March 2004 to December 2014. Cohorts were selected from two tertiary care (tygerberg academic hospital and red cross war memorial Children’s hospital) and two primary care (Gugulethu and Khayelitsha community health centres) facilities	Quantitative longitudinal analysis	Linking cohort data to the health information system data allowed efficient assessment of post-transfer outcomes

## Abbreviations

ART: Antiretroviral therapy
CBS: Case-based surveillance
CMIA: Client Management Information System
eHIS: Electronic Health Information System
EPMS: Electronic Patient Management System
EPTS: Electronic Patient Tracking System
HIS: Health information system
HPRN: Health patient registration number
HPRS: Health Patient Registration System
ID: Identity
IP: Implementing-partner
IPMS: Integrated Procurement Management System
MESH: Medical Subject Headings
NCDs: Non-communicable diseases
NHI: National Health Insurance
PEPFAR: The U.S. President’s Emergency Plan for AIDS Relief
PEPFAR-COP: PEPFAR Country Operational Plans
PHC: Primary Healthcare
PIMS: Patient Information Management System
PLHIV: People living with HIV
SADC: South African Development Community
SA: South Africa
TB: Tuberculosis
UNAIDS: The Joint United Nations Programme on HIV/AIDS (UNAIDS)
UPI: Unique Patient Identifier
WCP: Western Cape Province
WHO: World Health Organization
