# Supplementary material for: Progress towards unique patient identification and case-based surveillance within the Southern African development community
Source: Health Informatics J. Author manuscript; Available in PMC 2023 Jun 30. (PMC10311353; doi:10.1177/14604582221139058)
Supplement: Supplement [file NIHMS1907795-supplement-Supplement.pdf]

Appendix A: Assessment of progress towards CBS with a UPI – Botswana

**Person identification: assigning and using unique identifiers**	**Early**	Name-based record and/or aggregate data based on services, not people (tally sheets)	x	**Patient Information Management System (PIMS) is a facility based/clinic specific system with HIV and non-HIV data which makes use of a facility specific unique identifier (1) (in-country contact). There is partial implementation of the unique identifier; Botswana still has a combination of paper and electronic health information systems as most district hospitals and clinics are either connected to PIMS or are paper based (1). The goal is to move towards a national unique identifier using the OMANG number (1). Paper based records are aggregated at district and national level (2).**
	**Middle**	Unique identifiers at facility level	x	
	**Advanced**	Programme or national unique identifiers, people-centred health record systems	-	
**Investing in databases and interoperability**	**Early**	Low-cost paper-based record system/Traditional stationary costs	x	**Paper based records exist alongside PIMS; PIMS is also not integrated with the laboratory system, Integrated procurement management system (IPMS), results need to be manually transferred into PIMS (1). As PIMS is facility specific, identifiers differ across facilities (3). The goal is to move towards a national unique identifier using the OMANG number (1)**
	**Middle**	Facility-based electronic data systems/Basic computer / Open-access software	x	
	**Advanced**	Fully interoperable data system, linkage of information from multiple sources. Linkage with vital statistics, migration data. Useful for tracking individuals lost to follow up, etc	-	
**Confidentiality and security**	**Early**	Name-labelled paper files retained by the individual or kept under lock and key at facility	x	**Paper based records exist without mention of unique identifiers, PIMS uses a facility specific unique number; personal identifiers may be used for patient tracking between facilities (1).**
	**Middle**	Records coded with unique identifiers without personal content	-	
	**Advanced**	National system with health record data protected by law. Limited and enforced data access control	-	
**Data analysis, quality and use**	**Early**	Data officer transfers data from paper record into electronic health record or register, regular data quality reviews	x	**Lab results data has to be transferred into PIMS from IPIMS manually (1). Paper based records still exist, data is aggregated at district and national level (2). Difficulties tracking patients across facilities, due to incomplete data and different unique ids per facilities hamper accuracy of program level analysis and require manual intervention (1).**
	**Middle**	Programme or central-level analysis of data and creation of management dashboards, and other data analysis and reporting tools	-	
	**Advanced**	Local analyses of care and programmatic capacity. Standardized dashboards, data visualization and reports. Individual care facilitated by ease of data access, aggregation and review. Regular use of data for decision-making at individual, facility, programme and national levels	-	
**Transition from paper to electronic systems**	**Early**	Paper based record system. Records retained at facility or by individual	x	**Paper based records exist alongside electronic records (2). Labs data has to be transferred into PIMS from IPIMS manually, better linkage between facilities is required (1).**
	**Middle**	Offline electronic upload of data. On- or offline data access	x	
	**Advanced**	Fully online systems used across facilities, in community care. Links services within facility and across facilities	-	
**Sustainability of program improvements**	**Early**	Patient monitoring is only system in place to track individuals over time. Challenging to link individual data within and between facilities	x	**Facilities with the PIMS system have the ability to track a patient using the unique ID within a facility (1)(2)(3). Patients cannot be tracked across facilities and laboratory results are not automatically integrated into PIMS from IPMS (1) (in-country contact).**
	**Middle**	Limited ability to track individuals within a facility. Appointment scheduling, follow up within a facility. Within facility linkage of individual information from clinic to lab and pharmacy	x	
	**Advanced**	Individual records updated in real-time with clinical, lab, pharmacy and other data. Person-based records linked with death registry data	-	

Reference list

1. PEPFAR. Botswana Country Operational Plan (COP/ROP) 2019 Strategic Direction Summary. 2019;95. Available from: https://www.state.gov/wp-content/uploads/2019/09/Botswana_COP19-Strategic-Directional-Summary_public.pdf

2. Ndlovu K, Mogotlhwane T, Scott RE, Mars M. E-health interoperability landscape: Botswana. Proc 4th IASTED Int Conf Heal Informatics, AfricaHI 2016. 2016;(AfricaHI):202–7.

3. Farahani M, Price N, El-Halabi S, Mlaudzi N, Keapoletswe K, Lebelonyane R, et al. Impact of Health System Inputs on Health Outcome: A Multilevel Longitudinal Analysis of Botswana National Antiretroviral Program (2002-2013). PLoS One [Internet]. 2016;11(8):1–14. Available from: https://doi.org/10.1371/journal.pone.0160206

Appendix B: Assessment of progress towards CBS with a UPI – Eswatini (Swaziland)

**Person identification: assigning and using unique identifiers**	**Early**	Name-based record and/or aggregate data based on services, not people (tally sheets)	x	**The Client management information system (CMIS) is currently being rolled out nationwide; in facilities where the CMIS is implemented national ID numbers are used as unique identifiers; if the person does not have an ID they are given a medical card with a unique identifier to be used at all facilities. In facilities where the CMIS has not been implemented a manual paper-based record is used with unique id or ID number linked to the medical card; personal details such as name and surname captured into the CMIS (1) (in-country contact). For patients without national ID, CMIS creates a unique system generated identifier (2).**
	**Middle**	Unique identifiers at facility level	x	
	**Advanced**	Programme or national unique identifiers, people-centred health record systems	-	
**Investing in databases and interoperability**	**Early**	Low-cost paper-based record system/Traditional stationary costs	x	**CMIS is currently being rolled out nationwide however not all facility data syncs automatically and not all facilities have the CMIS in place (2). Facilities without the CMIS have a paper-based system (1). Eswatini currently has standalone information systems for laboratory (matured, currently proprietary), supply chain (under pilot, open source) and patient monitoring (2).**
	**Middle**	Facility-based electronic data systems/Basic computer / Open-access software	x	
	**Advanced**	Fully interoperable data system, linkage of information from multiple sources. Linkage with vital statistics, migration data. Useful for tracking individuals lost to follow up, etc	-	
**Confidentiality and security**	**Early**	Name-labelled paper files retained by the individual or kept under lock and key at facility	x	**Despite the use of a UPI personal content is captured into the medical record, patient name and surname details are captured into the CMIS (1) (3).**
	**Middle**	Records coded with unique identifiers without personal content	-	
	**Advanced**	National system with health record data protected by law. Limited and enforced data access control	-	
**Data analysis, quality and use**	**Early**	Data officer transfers data from paper record into electronic health record or register, regular data quality reviews	x	**When the CMIS is down data is captured on a paper form and then captured into the CMIS when the system is restored; this may often not occur due to staff workload resulting in incomplete data (2). For those using the medical card without an ID number, the card can often be lost and a new identifier assigned to a person, this makes tracking difficult and often results in duplicate records (2). Systems are currently standalone and need to be fully integrated to enable central level analysis (2).**
	**Middle**	Programme or central-level analysis of data and creation of management dashboards, and other data analysis and reporting tools	-	
	**Advanced**	Local analyses of care and programmatic capacity. Standardized dashboards, data visualization and reports. Individual care facilitated by ease of data access, aggregation and review. Regular use of data for decision-making at individual, facility, programme and national levels	-	
**Transition from paper to electronic systems**	**Early**	Paper based record system. Records retained at facility or by individual	x	**Synchronisation of data between facilities is slow (1). Paper based records still exist alongside the CMIS which needs to be updated manually when the system is down (PEPFAR, 2019b).**
	**Middle**	Offline electronic upload of data. On- or offline data access	x	
	**Advanced**	Fully online systems used across facilities, in community care. Links services within facility and across facilities	-	
**Sustainability of program improvements**	**Early**	Patient monitoring is only system in place to track individuals over time. Challenging to link individual data within and between facilities	x	**Patient tracking ability exists between facilities with the CMIS, however this is often hampered by duplicate records, incomplete data often due to staff workload impairing tracking between facilities and lack of integration with other systems (2).**
	**Middle**	Limited ability to track individuals within a facility. Appointment scheduling, follow up within a facility. Within facility linkage of individual information from clinic to lab and pharmacy	x	
	**Advanced**	Individual records updated in real-time with clinical, lab, pharmacy and other data. Person-based records linked with death registry data	-	

Reference list

1. Measure Evaluation. Implementing Swaziland's client management information system. Vol. 8. 2017
2. PEPFAR. Eswatini Country Operational Plan 2019 Strategic Direction Summary Table of Contents. 2019;2019(April)
3. Health Management Information Systems unit - Strategic Information Department (SID) of Ministry of Health Swaziland (2019) CMIS. Available at: http://www.hmisswaziland.com (Accessed: 20 September 2011).

Appendix C: Assessment of progress towards CBS with a UPI – Lesotho

**Person identification: assigning and using unique identifiers**	**Early**	Name-based record and/or aggregate data based on services, not people (tally sheets)	x	**Lesotho is currently rolling out the eregister program which includes a unique ART identifier for all people living with HIV allowing case monitoring (1). Facilities without the eregister are still paper based; paper-based records are clinic specific (1). There is still an over-reliance on paper-based monitoring systems (2).**
	**Middle**	Unique identifiers at facility level	x	
	**Advanced**	Programme or national unique identifiers, people-centred health record systems	-	
**Investing in databases and interoperability**	**Early**	Low-cost paper-based record system/Traditional stationary costs	x	**Paper and electronic record will be implemented side by side for a period until transition to electronic records only; potential for transfer from paper to electronic if the system is unavailable (1). Electronic medical records systems are however poorly implemented and there is an over-reliance on paper-based monitoring systems (2). Integration of all systems is reported as the next implementation step (1).**
	**Middle**	Facility-based electronic data systems/Basic computer / Open-access software	x	
	**Advanced**	Fully interoperable data system, linkage of information from multiple sources. Linkage with vital statistics, migration data. Useful for tracking individuals lost to follow up, etc	-	
**Confidentiality and security**	**Early**	Name-labelled paper files retained by the individual or kept under lock and key at facility	x	**Paper and electronic records implemented side by side for a period, paper files locked at facility (1) (in-country contact). Poor data systems, challenging to track patients across the continuum of care (3).**
	**Middle**	Records coded with unique identifiers without personal content	-	
	**Advanced**	National system with health record data protected by law. Limited and enforced data access control	-	
**Data analysis, quality and use**	**Early**	Data officer transfers data from paper record into electronic health record or register, regular data quality reviews	x	**Paper and electronic records are created simultaneously; if the electronic system is not available data will need to be transferred into the system manually when it is back up again (1). Incomplete data is still a challenge and impedes central level analysis (1).**
	**Middle**	Programme or central-level analysis of data and creation of management dashboards, and other data analysis and reporting tools	-	
	**Advanced**	Local analyses of care and programmatic capacity. Standardized dashboards, data visualization and reports. Individual care facilitated by ease of data access, aggregation and review. Regular use of data for decision-making at individual, facility, programme and national levels	-	
**Transition from paper to electronic systems**	**Early**	Paper based record system. Records retained at facility or by individual	x	**Paper and electronic records are created simultaneously; if the electronic system is not available data will need to be transferred into the system manually when it is back up again (1).**
	**Middle**	Offline electronic upload of data. On- or offline data access	x	
	**Advanced**	Fully online systems used across facilities, in community care. Links services within facility and across facilities	-	
**Sustainability of program improvements**	**Early**	Patient monitoring is only system in place to track individuals over time. Challenging to link individual data within and between facilities	x	**E-register can track patients throughout Lesotho in facilities where it has been implemented (1).**
	**Middle**	Limited ability to track individuals within a facility. Appointment scheduling, follow up within a facility. Within facility linkage of individual information from clinic to lab and pharmacy	x	
	**Advanced**	Individual records updated in real-time with clinical, lab, pharmacy and other data. Person-based records linked with death registry data	-	

Reference list

1. PEPFAR. Lesotho Country Operational Plan (COP/ROP) 2019 Strategic Direction Summary. 2019;95. Available from: https://www.state.gov/wp-content/uploads/2019/09/Lesotho_COP19-Strategic-Directional-Summary_public.pdf
2. Mugomeri, E., Olivier, D. and Heever, W. M. Van Den (2018) 'Health system challenges affecting the implementation of isoniazid preventive therapy in people living with HIV in Lesotho', HIV & AIDS Review, 17(4).
3. Avert.org (2019) HIV and AIDS in East and Southern Africa regional overview. Available at: https://www.avert.org/professionals/hiv-around-world/sub-saharan-africa/overview.

Appendix D: Assessment of progress towards CBS with a UPI – Mozambique

**Person identification: assigning and using unique identifiers**	**Early**	Name-based record and/or aggregate data based on services, not people (tally sheets)	x	**National Electronic patient tracking system (EPTS) in place in most PEPFAR sites c. Focus is on expanding use of unique identifiers to improve monitoring (1). Paper based record systems are still used as the main method for recording and tracking patient data; clinics are required to send standardized aggregate summary reports on important health indicators to the Ministry of Health (2). Although unique at the time of its issuance, an individual could have multiple NIDs due to subsequent entry into an existing health facility (e.g., patient lost NID) or other health care facilities (1).**
	**Middle**	Unique identifiers at facility level	-	
	**Advanced**	Programme or national unique identifiers, people-centred health record systems	-	
**Investing in databases and interoperability**	**Early**	Low-cost paper-based record system/Traditional stationary costs	x	**EPTS in the process of being implemented. Linkages to laboratory and medicine systems still need to be put in place (1). Paper based record systems are still used as the main method for recording and tracking patient data, with a lack of integration between various health information systems (2).**
	**Middle**	Facility-based electronic data systems/Basic computer / Open-access software	x	
	**Advanced**	Fully interoperable data system, linkage of information from multiple sources. Linkage with vital statistics, migration data. Useful for tracking individuals lost to follow up, etc	-	
**Confidentiality and security**	**Early**	Name-labelled paper files retained by the individual or kept under lock and key at facility	x	**Paper files maintained at the facility, EPTS is often only referred to if the patient paper file cannot be found at the facility (2). Unique identifiers implementation to be scaled up (1).**
	**Middle**	Records coded with unique identifiers without personal content	-	
	**Advanced**	National system with health record data protected by law. Limited and enforced data access control	-	
**Data analysis, quality and use**	**Early**	Data officer transfers data from paper record into electronic health record or register, regular data quality reviews	x	**EPTS data entry typically occurs the day after a patient's medical visit, posing challenges to data accuracy and completeness; aggregated data reporting (2). Focus is on expanding use of unique identifiers to improve monitoring (1)**
	**Middle**	Programme or central-level analysis of data and creation of management dashboards, and other data analysis and reporting tools	-	
	**Advanced**	Local analyses of care and programmatic capacity. Standardized dashboards, data visualization and reports. Individual care facilitated by ease of data access, aggregation and review. Regular use of data for decision-making at individual, facility, programme and national levels	-	
**Transition from paper to electronic systems**	**Early**	Paper based record system. Records retained at facility or by individual	x	**Paper based record systems are still used as the main method for recording and tracking patient data, patient data is entered into the EPTS at a later time, although there is a focus on scaling up EPTS implementation (1) (2).**
	**Middle**	Offline electronic upload of data. On- or offline data access	x	
	**Advanced**	Fully online systems used across facilities, in community care. Links services within facility and across facilities	-	
**Sustainability of program improvements**	**Early**	Patient monitoring is only system in place to track individuals over time. Challenging to link individual data within and between facilities	x	**Paper based record systems are still used as the main method for recording and tracking patient data, with a lack of integration between various health information systems (1). There is a need to improve community/facility linkages to track outcomes and patients lost to care (2)**
	**Middle**	Limited ability to track individuals within a facility. Appointment scheduling, follow up within a facility. Within facility linkage of individual information from clinic to lab and pharmacy	x	
	**Advanced**	Individual records updated in real-time with clinical, lab, pharmacy and other data. Person-based records linked with death registry data	-	

Reference list

1. PEPFAR (2019) 'Mozambique Country Operational Plan (COP/ROP) 2019 Strategic Direction Summary', p. 95. Available at: https://www.state.gov/wp-content/uploads/2019/09/Mozambique_COP19-Strategic-Directional-Summary_public.pdf.

2. Hochgesang, M. et al. (2018) 'Scaling-up health information systems to improve HIV treatment: An assessment of initial patient monitoring systems in Mozambique', International Journal of Med Inform, (97), pp. 322–330. doi: 10.1016/j.ijmedinf.2016.11.002.Scaling-up.

Appendix E: Assessment of progress towards CBS with a UPI – Namibia

**Person identification: assigning and using unique identifiers**	**Early**	Name-based record and/or aggregate data based on services, not people (tally sheets)	x	**PEPFAR is in the process of introducing a unique health identifier across disease areas. There is a unique ART number which has the potential to enable case-based surveillance on the Electronic Patient Management System (ePMS). Aggregated data is reported monthly (1).**
	**Middle**	Unique identifiers at facility level	x	
	**Advanced**	Programme or national unique identifiers, people-centred health record systems	-	
**Investing in databases and interoperability**	**Early**	Low-cost paper-based record system/Traditional stationary costs	x	**Mostly paper-based system, with the exception of ePMS for HIV (2). All systems capable of capturing the ART number, but challenges with actually capturing/correct capturing of ART number (2) (3). Systems are not integrated, data from the electronic dispensing tool (EDT) and ePMS need to be manually collected and aggregated for reporting (1).**
	**Middle**	Facility-based electronic data systems/Basic computer / Open-access software	x	
	**Advanced**	Fully interoperable data system, linkage of information from multiple sources. Linkage with vital statistics, migration data. Useful for tracking individuals lost to follow up, etc	-	
**Confidentiality and security**	**Early**	Name-labelled paper files retained by the individual or kept under lock and key at facility	x	**Paper files do not currently have a unique identifier and are locked at the facility (3). ePMS for HIV - all systems capable of capturing the ART number, but challenges with actually capturing/correct capturing of ART number (2)(3).**
	**Middle**	Records coded with unique identifiers without personal content	-	
	**Advanced**	National system with health record data protected by law. Limited and enforced data access control	-	
**Data analysis, quality and use**	**Early**	Data officer transfers data from paper record into electronic health record or register, regular data quality reviews	x	**Patient information collected on paper-based forms and transferred into ePMS (1). While systems are not yet integrated, incorrect ART numbers further limits data analysis ability, results in double counting (2).**
	**Middle**	Programme or central-level analysis of data and creation of management dashboards, and other data analysis and reporting tools	-	
	**Advanced**	Local analyses of care and programmatic capacity. Standardized dashboards, data visualization and reports. Individual care facilitated by ease of data access, aggregation and review. Regular use of data for decision-making at individual, facility, programme and national levels	-	
**Transition from paper to electronic systems**	**Early**	Paper based record system. Records retained at facility or by individual	x	**Patient information collected on paper based forms and transferred into ePMS(1). Paper records remain at the facility (2).**
	**Middle**	Offline electronic upload of data. On- or offline data access	x	
	**Advanced**	Fully online systems used across facilities, in community care. Links services within facility and across facilities	-	
**Sustainability of program improvements**	**Early**	Patient monitoring is only system in place to track individuals over time. Challenging to link individual data within and between facilities	x	**ePMS has the functionality to track patients, however systems and facilities still need to be integrated and challenges with capturing of the ART number persist (2). Aggregated data is reported monthly through manual collection of data from different systems (1).**
	**Middle**	Limited ability to track individuals within a facility. Appointment scheduling, follow up within a facility. Within facility linkage of individual information from clinic to lab and pharmacy	x	
	**Advanced**	Individual records updated in real-time with clinical, lab, pharmacy and other data. Person-based records linked with death registry data	-	

Reference list

1. Agolory, S. et al. (2018) 'Low case finding among men and poor viral load suppression among adolescents are impeding Namibia's ability to achieve UNAIDS 90-90-90 targets', Open Forum Infectious Diseases, 5(9), pp. 1–8. doi: 10.1093/ofid/ofy200.
2. PEPFAR. Namibia Country Operational Plan (COP/ROP) 2019 Strategic Direction Summary. 2019;95. Available from: https://www.state.gov/wp-content/uploads/2019/09/Namibia_COP19-Strategic-Directional-Summary_public.pdf
3. Nengomasha, C. T. et al. (2018) 'Health information systems in Namibia', Information and Learning Science, 119(7–8), pp. 358–376. doi: 10.1108/ILS-03-2018-0015.

Appendix F: Assessment of progress towards CBS with a UPI – South Africa

**Person identification: assigning and using unique identifiers**	**Early**	Name-based record and/or aggregate data based on services, not people (tally sheets)	x	**South Africa has begun implementing a Health Patient Registration System (HPRS) for the creation of longitudinal health records; this system uses the national identification (ID) number or other legal person identification (e.g. passports) as a national unique identifier (National Department of Health South Africa, 2019). Improving the use of unique identifiers across sites and programs in clinical settings is however necessary (1).**
	**Middle**	Unique identifiers at facility level	x	
	**Advanced**	Programme or national unique identifiers, people-centred health record systems	-	
**Investing in databases and interoperability**	**Early**	Low-cost paper-based record system/Traditional stationary costs	x	**Existing health information systems in South Africa are predominantly paper-based; patient data is still manually recorded in the patient's file, while data required for monitoring and evaluation purposes is hand written by the nurses in registers (2).<br>HPRS implementation with a UPI has not reached scale as yet (1). The integration of the HIV health system TIER.Net with other key databases such as the national laboratory system using the unique identifier is a critical next step (1).**
	**Middle**	Facility-based electronic data systems/Basic computer / Open-access software	x	
	**Advanced**	Fully interoperable data system, linkage of information from multiple sources. Linkage with vital statistics, migration data. Useful for tracking individuals lost to follow up, etc	-	
**Confidentiality and security**	**Early**	Name-labelled paper files retained by the individual or kept under lock and key at facility	x	**Research conducted as part of this study has shown that name labelled paper files are still predominantly used.**
	**Middle**	Records coded with unique identifiers without personal content	-	
	**Advanced**	National system with health record data protected by law. Limited and enforced data access control	-	
**Data analysis, quality and use**	**Early**	Data officer transfers data from paper record into electronic health record or register, regular data quality reviews	x	**Data required for monitoring and evaluation purposes is hand written by the nurses in registers, manual calculations are uploaded into the District health information system (DHIS) for key indicators for aggregated monitoring (2).**
	**Middle**	Programme or central-level analysis of data and creation of management dashboards, and other data analysis and reporting tools	-	
	**Advanced**	Local analyses of care and programmatic capacity. Standardized dashboards, data visualization and reports. Individual care facilitated by ease of data access, aggregation and review. Regular use of data for decision-making at individual, facility, programme and national levels	-	
**Transition from paper to electronic systems**	**Early**	Paper based record system. Records retained at facility or by individual	x	**Data required for monitoring and evaluation purposes is hand written by the nurses in registers, manual calculations are uploaded into the District health information system (DHIS) (2).**
	**Middle**	Offline electronic upload of data. On- or offline data access	x	
	**Advanced**	Fully online systems used across facilities, in community care. Links services within facility and across facilities	-	
**Sustainability of program improvements**	**Early**	Patient monitoring is only system in place to track individuals over time. Challenging to link individual data within and between facilities	x	**Implementation and use of the unique identifier across sites still need to be improved, to enable tracking of patients through the health system (1).**
	**Middle**	Limited ability to track individuals within a facility. Appointment scheduling, follow up within a facility. Within facility linkage of individual information from clinic to lab and pharmacy	x	
	**Advanced**	Individual records updated in real-time with clinical, lab, pharmacy and other data. Person-based records linked with death registry data	-	

Reference list

1. PEPFAR. South Africa Country Operational Plan Strategic Direction Summary [Internet]. 2019. Available from: https://www.state.gov/wp-content/uploads/2019/09/South-Africa_COP19-Strategic-Directional-Summary_public.pdf
2. Wright, G., Mahony, D. O. and Cilliers, L. (2017) 'Electronic health information systems for public health care in South Africa : a review of current operational systems 10 th Health Informatics in Africa Conference (HELINA 2017)
3. Electronic health information systems for public health care in South Africa : a review of current operational systems', (January). doi: 10.12856/JHIA-2017-v4-i1-164.

Appendix G: Assessment of progress towards CBS with a UPI – Zambia

**Person identification: assigning and using unique identifiers**	**Early**	Name-based record and/or aggregate data based on services, not people (tally sheets)	X	**The SmartCare system for HIV has programme specific identifiers, but this has not been implemented in all facilities (1). Data from each visit are copied to a local database and to a portable SmartCard that is retained by patients; this dual data collection system allows transfer of an individual's medical record across facilities (2). Data is aggregated on a monthly basis (2).**
	**Middle**	Unique identifiers at facility level	X	
	**Advanced**	Programme or national unique identifiers, people-centred health record systems	-	
**Investing in databases and interoperability**	**Early**	Low-cost paper-based record system/Traditional stationary costs	X	**In facilities with access to Smartcare the implementing partners (IPs) are responsible for data entry into the system from paper-based forms which have information collected by clinicians (3). In more rural areas information is captured on SmartCare forms and the forms transported to an area with internet connectivity for data to be captured (3). Paper-based system exists alongside the electronic system. Laboratory data has not been integrated into the Smartcare system (1).**
	**Middle**	Facility-based electronic data systems/Basic computer / Open-access software	X	
	**Advanced**	Fully interoperable data system, linkage of information from multiple sources. Linkage with vital statistics, migration data. Useful for tracking individuals lost to follow up, etc	-	
**Confidentiality and security**	**Early**	Name-labelled paper files retained by the individual or kept under lock and key at facility	X	**While records are coded with the unique identifier from the Smartcare card, personal data is also collected in case the card is lost to enable access to the patient record (2). Data from each visit are copied to a local database and to a portable SmartCard that is retained by patients; this dual data collection system allows transfer of an individual's medical record across facilities (2).**
	**Middle**	Records coded with unique identifiers without personal content	-	
	**Advanced**	National system with health record data protected by law. Limited and enforced data access control	-	
**Data analysis, quality and use**	**Early**	Data officer transfers data from paper record into electronic health record or register, regular data quality reviews	X	**Some clinics may have poor internet connectivity so patient health information is captured on paper and transferred into the system at a location with internet connectivity (4). The implementing partners (IPs) are responsible for data entry mainly from paper-based forms which have information collected by clinicians, which is then entered into a computer (3). Data is aggregated on a monthly basis (2).**
	**Middle**	Programme or central-level analysis of data and creation of management dashboards, and other data analysis and reporting tools	-	
	**Advanced**	Local analyses of care and programmatic capacity. Standardized dashboards, data visualization and reports. Individual care facilitated by ease of data access, aggregation and review. Regular use of data for decision-making at individual, facility, programme and national levels	-	
**Transition from paper to electronic systems**	**Early**	Paper based record system. Records retained at facility or by individual	X	**Data from each visit are copied to a local database and to a portable SmartCard that is retained by patients; this dual data collection system allows manual transfer of an individual's medical record across facilities (2). Data uploads from paper forms required for data continuity and completeness (1).**
	**Middle**	Offline electronic upload of data. On- or offline data access	X	
	**Advanced**	Fully online systems used across facilities, in community care. Links services within facility and across facilities	-	
**Sustainability of program improvements**	**Early**	Patient monitoring is only system in place to track individuals over time. Challenging to link individual data within and between facilities	X	**System strengthening and integration is required for better patient tracking and monitoring capabilities (1). Reporting data is based on aggregated information (3).**
	**Middle**	Limited ability to track individuals within a facility. Appointment scheduling, follow up within a facility. Within facility linkage of individual information from clinic to lab and pharmacy		
	**Advanced**	Individual records updated in real-time with clinical, lab, pharmacy and other data. Person-based records linked with death registry data		

Reference list

1. PEPFAR. PEPFAR Zambia Country Operational Plan (COP) 2019 Strategic Direction Summary April 12, 2019. 2019; Available from: https://www.state.gov/wp-content/uploads/2019/09/Zambia_COP19-Strategic-Directional-Summary_public.pdf
2. Moucheraud, C. et al. (2017) 'Sustainability of health information systems: A three-country qualitative study in southern Africa', BMC Health Services Research. BMC Health Services Research, 17(1), pp. 1–11. doi: 10.1186/s12913-016-1971-8.
3. Gumede-Moyo, S. et al. (2019) 'A qualitative inquiry into implementing an electronic health record system (SmartCare) for prevention of mother-to-child transmission data in Zambia: A retrospective study', BMJ Open, 9(9), pp. 1–9. doi: 10.1136/bmjopen-2019-030428.
4. Munthali, T. et al. (2017) 'Underutilisation of routinely collected data in the HIV programme in Zambia: A review of quantitatively analysed peer-reviewed articles', Health Research Policy and Systems. Health Research Policy and Systems, 15(1), pp. 1–10. doi: 10.1186/s12961-017-0221-9.

Appendix H: Assessment of progress towards CBS with a UPI – Zimbabwe

**Person identification: assigning and using unique identifiers**	**Early**	Name-based record and/or aggregate data based on services, not people (tally sheets)	X	**PEPFAR currently engaging and supporting the MOH in Zimbabwe to develop unique identifier code for PLHIV (1). There are limited facilities that are currently using the Electronic Patient Management System (EPMS) and no unique identifiers to address client duplication with a lot of clients captured as lost to follow up as a result (2).**
	**Middle**	Unique identifiers at facility level	-	
	**Advanced**	Programme or national unique identifiers, people-centred health record systems	-	
**Investing in databases and interoperability**	**Early**	Low-cost paper-based record system/Traditional stationary costs	X	**Reliance on facility base paper health information systems (2). There is a need to improve the data linkage between facilities and community structures (community cadres, IPs) as referrals and other out of facility services are not fully captured at facility level, particularly in the absence of a paper referral slips (2).**
	**Middle**	Facility-based electronic data systems/Basic computer / Open-access software	-	
	**Advanced**	Fully interoperable data system, linkage of information from multiple sources. Linkage with vital statistics, migration data. Useful for tracking individuals lost to follow up, etc	-	
**Confidentiality and security**	**Early**	Name-labelled paper files retained by the individual or kept under lock and key at facility	X	**Tracking and referral systems mostly manual and paper based with no unique identifiers (1). There is a need to improve the data linkage between facilities (2).**
	**Middle**	Records coded with unique identifiers without personal content	-	
	**Advanced**	National system with health record data protected by law. Limited and enforced data access control	-	
**Data analysis, quality and use**	**Early**	Data officer transfers data from paper record into electronic health record or register, regular data quality reviews	X	**Limited data personnel capable of capturing data into the EPMS system at facilities (2).**
	**Middle**	Programme or central-level analysis of data and creation of management dashboards, and other data analysis and reporting tools	-	
	**Advanced**	Local analyses of care and programmatic capacity. Standardized dashboards, data visualization and reports. Individual care facilitated by ease of data access, aggregation and review. Regular use of data for decision-making at individual, facility, programme and national levels	-	
**Transition from paper to electronic systems**	**Early**	Paper based record system. Records retained at facility or by individual	X	**Record, tracking and referral systems remain paper based (1). Systems require data to be uploaded by personnel (2).**
	**Middle**	Offline electronic upload of data. On- or offline data access	-	
	**Advanced**	Fully online systems used across facilities, in community care. Links services within facility and across facilities	-	
**Sustainability of program improvements**	**Early**	Patient monitoring is only system in place to track individuals over time. Challenging to link individual data within and between facilities	X	**There is a need to improve the data linkage between facilities (2)**
	**Middle**	Limited ability to track individuals within a facility. Appointment scheduling, follow up within a facility. Within facility linkage of individual information from clinic to lab and pharmacy		
	**Advanced**	Individual records updated in real-time with clinical, lab, pharmacy and other data. Person-based records linked with death registry data		

Reference list
1. PEPFAR, 'PEPFAR Zimbabwe Country Operational Plan 2019 Strategic Direction Summary', p. 80. Available at: http://www.pepfar.gov/documents/organization/250290.pdf.
2. Health Gap Global Access Project. COP20 COMMUNITY ZIMBABWE [Internet]. Vol. 22, Community Priorities. 2020. Available from: https://healthgap.org/wp-content/uploads/2020/02/Community-COP20-Zimbabwe.pdf
